# Advances in the Management of Pediatric Inflammatory Bowel Disease: From Biologics to Small Molecules

**DOI:** 10.3390/ph19010176

**Published:** 2026-01-20

**Authors:** Benedetta Mucci, Elisabetta Palazzolo, Flaminia Ruberti, Lorenzo Ientile, Marco Natale, Susanna Esposito

**Affiliations:** Pediatric Clinic, Department of Medicine and Surgery, University Hospital of Parma, 43126 Parma, Italy; benedetta.mucci@unipr.it (B.M.); elisabetta.palazzolo@unipr.it (E.P.); flaminia.ruberti@unipr.it (F.R.); lorenzo.ientile@unipr.it (L.I.); marco.natale@unipr.it (M.N.)

**Keywords:** pediatric inflammatory bowel disease, biologics, small molecules, anti-TNF, JAK inhibitors, sphingosine-1-phosphate modulators, precision medicine

## Abstract

**Background:** The management of pediatric inflammatory bowel disease (PIBD) has evolved significantly over the past two decades, transitioning from corticosteroids and immunomodulators to biologic and small-molecule therapies. These advances have aimed not only to control inflammation but also to promote mucosal healing, improve growth, and enhance long-term quality of life. **Objectives:** This narrative review summarizes current evidence on the efficacy, safety, and clinical applications of biologic and novel small-molecule therapies in PIBD, highlighting emerging trends in personalized and precision-based management. **Methods:** A literature search was performed across PubMed, Embase, and the Cochrane Library, focusing on studies published within the last five years. Additional data were retrieved from key guidelines and position papers issued by ECCO–ESPGHAN, SIGENP, the FDA, and the EMA. **Results:** Anti–tumor necrosis factor (TNF) agents such as infliximab and adalimumab remain first-line biologics with proven efficacy in remission induction and maintenance. Newer biologics—vedolizumab, ustekinumab, risankizumab, and mirikizumab—offer alternatives for anti-TNF-refractory cases, showing encouraging short-term results and favorable safety profiles. Although many are approved only for adults with limited pediatric evidence, emerging small molecules—including Janus kinase (JAK) inhibitors (tofacitinib, upadacitinib) and sphingosine-1-phosphate (S1P) modulators (etrasimod)—provide oral, rapidly acting, and non-immunogenic treatment options for refractory disease. Furthermore, the gut microbiome is increasingly recognized as an emerging therapeutic target in PIBD, with growing evidence that host–microbiome interactions can influence both the efficacy and safety of biologics and small-molecule therapies. **Conclusions:** While biologics and small molecules have transformed PIBD management, challenges remain, including high treatment costs, limited pediatric trial data, and variable access worldwide. Future directions include multicenter pediatric studies, integration of pharmacogenomics, and biomarker-guided precision medicine to optimize early, individualized treatment and improve long-term outcomes.

## 1. Introduction

Inflammatory bowel disease (IBD), encompassing Crohn’s disease (CD) and ulcerative colitis (UC), is a chronic, relapsing, and potentially life-altering condition characterized by inflammation of the gastrointestinal tract, most commonly affecting the small intestine and colon [1]. Contemporary evidence indicates that its etiology is multifactorial, arising from a complex interplay among genetic susceptibility, environmental exposures, epithelial barrier dysfunction, and dysregulated immune responses. Increasing attention has been directed toward the gut microbiome, whose composition and metabolic activity strongly influence mucosal immunity. Dysbiosis—marked by reduced microbial diversity, loss of beneficial commensals, and expansion of pro-inflammatory taxa—has been shown to contribute directly to IBD pathogenesis by promoting aberrant immune activation and impairing epithelial homeostasis, as well as modulating responsiveness to medical therapy [2].

Approximately 25% of IBD cases are diagnosed during childhood or adolescence. Pediatric-onset IBD (PIBD) is frequently associated with a more extensive and severe disease phenotype than adult-onset forms and can be complicated by growth failure, delayed puberty, increased risk of malignancy, and adverse effects on school performance and health-related quality of life [1]. These unique developmental and psychosocial challenges underscore the need for early, effective, and individualized intervention strategies.

Historically, corticosteroids (CS) have served as the cornerstone for inducing remission in IBD [1]. However, their prolonged use is associated with significant adverse outcomes, particularly growth suppression and impaired bone health. Consequently, minimizing steroid exposure and achieving CS-free remission have become central therapeutic goals. Traditional immunomodulators such as azathioprine (AZA) and methotrexate (MTX) have been used to maintain remission, but their efficacy is limited and adverse events are not uncommon [3].

The introduction of biologic therapies has revolutionized the management of adult and pediatric IBD. Infliximab (IFX), the first monoclonal antibody targeting tumor necrosis factor-alpha (TNF-α), demonstrated substantial efficacy and paved the way for the development of additional biologic agents aimed at specific inflammatory pathways. Despite their success, biologics are limited by high costs, variable treatment response, and the risk of immunogenicity, creating disparities in access—particularly in pediatric populations. In this context, biosimilars, therapeutic optimization strategies such as proactive therapeutic drug monitoring (TDM), and emerging drug-delivery innovations have become crucial for improving the affordability and equitable use of biologic therapies in children [4].

As the field moves toward precision and personalized medicine, treatment paradigms increasingly incorporate biomarker-guided approaches, pharmacogenomics, and—more recently—microbiome-informed strategies to optimize therapy selection and maximize the likelihood of achieving deep remission while minimizing toxicity [5]. However, significant knowledge gaps remain regarding predictors of treatment response, the optimal sequencing of advanced therapies, and the integration of emerging microbiome and immune biomarkers into clinical decision-making.

The aim of this narrative review is to provide an updated and comprehensive overview of the indications, efficacy, and adverse events associated with biologic therapies for PIBD, encompassing both established and emerging agents, while highlighting current knowledge gaps and future directions in personalized and precision-based care.

## 2. Methods

This study was conducted as a narrative review aimed at synthesizing and contextualizing current evidence on biologic and small-molecule therapies in PIBD. A narrative approach was intentionally selected because the objectives extended beyond quantitative comparison of outcomes to include interpretation of evolving treatment paradigms, integration of international guideline recommendations, and discussion of emerging real-world pediatric data—elements that are not optimally addressed through a formal systematic review or meta-analytic methodology. Accordingly, the review did not follow PRISMA reporting standards, and no quantitative synthesis was planned.

A structured but non-systematic literature search was performed using PubMed, Embase, and the Cochrane Library. The search primarily targeted publications from the last five years to capture recent therapeutic developments. However, earlier landmark trials and pivotal studies published between 2006 and 2025 were deliberately included when they provided foundational evidence for established therapies (e.g., infliximab, adalimumab) or were necessary to contextualize current treatment algorithms. The final temporal scope was therefore defined as key pediatric and adult studies published between 2006 and 2025, with particular emphasis on literature from 2020 onward.

Search terms combined concepts related to inflammatory bowel disease, biologic therapies, small molecules, and pediatric populations (e.g., IBD AND biologics/small molecules AND pediatrics). For readability, the full electronic search strings are not reproduced.

### 2.1. Inclusion and Exclusion Criteria

At the title and abstract screening stage, records were excluded if they:

- were unrelated to inflammatory bowel disease;
- did not address biologic or small-molecule therapies;
- lacked pediatric data or clear relevance to pediatric clinical practice;
- were not published in English; or
- consisted of article types without original or synthesized scientific content (e.g., editorials, opinion pieces, commentaries).

Full-text articles were subsequently reviewed and included when they met the following criteria:

- relevance to the clinical management of PIBD;
- contribution to understanding efficacy, safety, dosing, treatment positioning, or real-world use of biologic or small-molecule therapies; and
- sufficient methodological rigor to support interpretation of findings in a clinical context.

Because this was a narrative review, no predefined numerical thresholds, formal risk-of-bias tools, or scoring systems were applied, and the number of excluded studies was not systematically recorded. Consequently, a PRISMA-style flow diagram was not generated.

### 2.2. Assessment of Methodological Quality

Methodological quality was assessed qualitatively rather than formally, in keeping with the narrative design. Priority was given to:

- randomized controlled trials and well-designed prospective studies when available;
- large multicenter cohorts and registry-based studies for pediatric real-world evidence;
- studies with clearly defined patient populations, outcomes, and follow-up; and
- consistency of findings with existing guidelines and consensus recommendations.

When pediatric randomized data were lacking, high-quality adult trials were included if their findings were considered biologically plausible and clinically informative for pediatric practice, with explicit acknowledgment of extrapolation. Studies with major methodological limitations, unclear outcome definitions, or minimal relevance to contemporary PIBD management were deprioritized or excluded.

### 2.3. Guidelines and Consensus Documents

To complement the database search, guidelines, consensus statements, and position papers from major professional and regulatory bodies were reviewed. ECCO–ESPGHAN and SIGENP documents were prioritized because they provide the most comprehensive pediatric-specific recommendations relevant to European practice. However, guidance and regulatory perspectives from NASPGHAN, Canadian societies, the FDA, and the EMA were also incorporated when pertinent, ensuring an international and balanced perspective.

This combined approach allowed integration of peer-reviewed evidence with expert consensus and regulatory insight, aligning with the objectives and methodological boundaries of a narrative review.

## 3. Anti-TNF Therapy in Pediatric Patients with Inflammatory Bowel Disease

Numerous studies have identified TNFα as a pivotal pro-inflammatory cytokine involved in the pathogenesis and perpetuation of IBD. Elevated TNFα concentrations have been consistently detected in the serum and stool of patients with both CD and UC [6].

This evidence has established TNFα as a prime therapeutic target, leading to the development of anti-TNFα biologic agents. The first drugs introduced in this class were infliximab and adalimumab. Infliximab is a chimeric monoclonal antibody, comprising human and murine IgG components, and has been widely employed in the management of multiple autoimmune and autoinflammatory conditions [7]. Conversely, adalimumab is a fully human recombinant IgG monoclonal antibody [8]. Both agents exert their therapeutic effect by binding with high affinity to soluble and membrane-bound TNFα, thereby preventing its interaction with cell-surface receptors and effectively inhibiting downstream pro-inflammatory signaling [9].

The standard infliximab regimen consists of an induction phase with intravenous infusions of 5 mg/kg at weeks 0, 2, and 6, followed by maintenance therapy with 5 mg/kg every 8 weeks. In certain clinical scenarios—such as in children weighing less than 30 kg, those with extensive disease involvement, or patients presenting with hypoalbuminemia—higher induction doses (e.g., 10 mg/kg) or shortened dosing intervals may be required to achieve and maintain therapeutic trough concentrations [10].

Adalimumab, administered via subcutaneous injection, follows a weight-based dosing protocol. For patients weighing ≥40 kg, the induction regimen consists of 160 mg initially, followed by 80 mg two weeks later, and maintenance dosing of 40 mg every two weeks. For patients weighing <40 kg, the induction doses are 80 mg, followed by 40 mg after two weeks, with a maintenance dose of 20 mg every two weeks. In patients demonstrating loss of response or subtherapeutic trough levels, dose escalation or interval shortening may be necessary to restore clinical remission [11].

Since their approval, anti-TNF biologics have progressively transformed the therapeutic landscape of PIBD, evolving from adjunctive options to cornerstone treatments in achieving and maintaining long-term remission.

Table 1 summarizes anti-TNF drugs approved for pediatric patients with IBD.

Despite the extensive clinical experience with anti-TNF agents in PIBD, several important limitations must be acknowledged. First, much of the evidence supporting infliximab and adalimumab use in children is extrapolated from adult studies, with relatively few pediatric randomized controlled trials available. As a result, key aspects of pediatric care—including optimal dosing in younger or low-weight patients, long-term safety, and predictors of durable remission—remain incompletely defined. Second, dose escalation, combination therapy, and proactive therapeutic drug monitoring, although commonly applied in clinical practice, are supported largely by retrospective cohorts and real-world registries rather than prospective pediatric trials. This reliance on observational data introduces risks of selection bias and heterogeneity in treatment approaches. Additionally, several therapeutic strategies frequently used in children, such as higher induction doses or interval shortening for infliximab, represent off-label applications that lack formal regulatory approval, underscoring the need for child-specific pharmacokinetic and pharmacodynamic data. Finally, limited data exist on long-term outcomes beyond adolescence, including malignancy risk, immunogenicity trajectories, and the consequences of prolonged biologic exposure during critical windows of growth and development. These gaps highlight the pressing need for well-designed, multicenter pediatric trials to strengthen the evidence base guiding anti-TNF therapy in children with IBD.

### 3.1. Infliximab and Adalimumab in Pediatric Crohn’s Disease

In earlier versions of the pediatric CD management guidelines, both the ECCO and the ESPGHAN recommended the use of anti-TNFα agents as second-line therapy. These biologics were to be introduced only after the failure of conventional treatments such as exclusive enteral nutrition (EEN), CS, 5-aminosalicylic acid (5-ASA), and immunomodulators (e.g., azathioprine [AZA] and methotrexate [MTX]), in accordance with the traditional “step-up” therapeutic approach [12,13].

However, accumulating evidence supporting the efficacy and safety of early biologic intervention is driving a paradigm shift toward earlier use of anti-TNFα agents. For instance, in a prospective study of 191 propensity score–matched pediatric CD patients, Kugathasan et al. demonstrated that early administration of anti-TNFα therapy (within 12 weeks of diagnosis) significantly reduced the risk of penetrating complications, although it did not affect the development of stricturing disease [14].

Similarly, a meta-analysis of 13 studies—including two randomized controlled trials and eleven cohort studies involving 861 children and young adults—found that early biologic initiation was associated with higher rates of clinical remission, fewer relapses, improved mucosal healing, and better linear growth outcomes compared with delayed biologic use or conventional treatment [15].

The TISKIDS trial represents a landmark head-to-head comparison between a top-down approach using infliximab and standard first-line therapies (EEN or CS) in pediatric patients with moderate-to-severe CD. At 52 weeks, 41% of children receiving infliximab as first-line therapy achieved clinical remission, compared with only 12% in the conventional treatment group [16].

Reflecting these findings, the most recent ECCO–ESPGHAN guidelines (2021) [17] recommend early initiation of biologic therapy at diagnosis for children with extensive disease, deep colonic ulcerations, perianal involvement, stricturing or penetrating behavior, or growth failure. Anti-TNFα agents also remain endorsed as second-line treatments for children with moderate-to-severe CD unresponsive to standard therapies [17]. In alignment, the Asia Pan-Pacific Society for Pediatric Gastroenterology, Hepatology, and Nutrition (APPSPGHAN) updated its 2022 recommendations, advocating early biologic intervention in cases of severe luminal or perianal disease [18].

### 3.2. Infliximab and Adalimumab in Pediatric Ulcerative Colitis

According to the ECCO–ESPGHAN guidelines [17], infliximab is the recommended first-line biologic therapy for children with chronically active, moderate-to-severe UC unresponsive to conventional treatments, as well as for those with acute severe UC (ASUC) showing insufficient response after five days of intravenous corticosteroids.

The efficacy of infliximab in pediatric UC has been consistently demonstrated across multiple studies. In an early pivotal study (2008), approximately 70% of pediatric patients with UC responded to infliximab, with a marked reduction in the need for colectomy among responders and a favorable safety profile [19]. Larger cohort analyses later reported steroid-free remission rates of 38% at 12 months and 21% at 24 months, alongside a 61% reduction in colectomy rates at two years [20].

A multicentric Polish study involving 42 children aged 4–18 years with varying disease activity further confirmed infliximab’s efficacy in preventing early colectomy. Following induction therapy, 33.3% achieved clinical response and 26.2% achieved remission, while two patients required surgery and two experienced anaphylactic reactions. After maintenance therapy, 57.1% remained in clinical remission, although three children required colectomy [21].

In a landmark multicenter trial by Hyams et al. (2012) [22], infliximab induced a clinical response at week 8 in 73.3% of pediatric patients with moderate-to-severe UC unresponsive to conventional therapy. The overall remission rate at week 54 was 28.6%, with the highest rates observed in the every-8-week (q8w) maintenance group [22].

While adalimumab is not currently recommended as a first-line biologic for pediatric UC in European, Asian, or Canadian guidelines, several studies have confirmed its efficacy and safety [23]. In the ENVISION I trial, 93 children with moderate-to-severe UC were randomized to high-dose adalimumab, maintenance dosing, or placebo. The study demonstrated significant clinical remission and response rates with a favorable safety profile. Consequently, the ECCO–ESPGHAN guidelines now recommend adalimumab for pediatric patients with intolerance or secondary loss of response (LOR) to infliximab, guided by therapeutic drug monitoring (TDM), but not for those with primary non-response [23].

Expanding on these findings, real-world registry data from the SIGENP-IBD group (n = 32; median age ≈ 10 years) involving children previously treated with infliximab (due to intolerance or non-response) reported that approximately 41% achieved CS-free remission and 28% achieved mucosal healing at 52 weeks. The relapse-free probability was 69% at 12 weeks, 59% at 30 weeks, and 53% at one year [24].

An ongoing clinical challenge remains the reduced efficacy of adalimumab following infliximab failure in pediatric UC. Evidence indicates that anti-TNF–experienced patients respond less favorably than anti-TNF–naïve individuals, reinforcing the guideline recommendation that adalimumab should not be used in cases of primary non-response to infliximab, but may be appropriate for those with secondary LOR or intolerance [23].

Finally, a major limitation in the use of biologics for pediatric UC is their high cost, particularly in low-resource settings. The introduction of biosimilar agents has alleviated this barrier to some extent, as multiple studies have demonstrated that biosimilars are comparable in efficacy, safety, and immunogenicity to originator biologics [25].

### 3.3. Combination Therapy

The ECCO–ESPGHAN guidelines recommend initiating infliximab in combination with an immunomodulator for the treatment of pediatric CD. Discontinuation of the immunomodulator may be considered after 6–12 months in patients who have achieved optimal therapeutic drug concentrations and sustained clinical remission [12,26].

In pediatric populations, evidence directly comparing combination therapy and monotherapy remains limited, with most data derived from retrospective studies. These studies generally indicate that combination therapy is associated with improved long-term durability of infliximab response and a higher probability of treatment continuation at five years compared with monotherapy [27,28]. However, the only prospective, open-label randomized controlled trial in children found no significant advantage in extending combination therapy to 54 weeks versus discontinuing the immunomodulator after 26 weeks [29].

By contrast, evidence supporting adalimumab-based combination therapy is less robust. A post hoc analysis of the IMAgINE-1 trial, a pediatric randomized controlled study, demonstrated no significant difference in clinical remission rates between patients receiving adalimumab plus a thiopurine or methotrexate and those receiving adalimumab monotherapy (36% vs. 30%) [30]. Similarly, data from the PANTS cohort study supported these findings, showing no improvement in clinical outcomes with combination therapy, although patients receiving combined treatment exhibited a lower incidence of anti-adalimumab antibody formation [31].

Consequently, the ECCO–ESPGHAN guidelines do not recommend routine combination therapy when adalimumab is used as the first anti-TNF agent. Nonetheless, combination therapy may be considered appropriate for patients previously exposed to infliximab or for those at high risk of immunogenicity or treatment failure when initiating adalimumab [12].

### 3.4. Therapeutic Drug Monitoring and Dose Optimization

Approximately 10–30% of patients receiving anti-TNF therapy fail to achieve an initial clinical response, a condition known as primary non-response. Furthermore, up to 50% of initial responders may experience a subsequent secondary LOR over time. Both types of treatment failure are frequently associated with subtherapeutic trough drug concentrations, the development of ADA, or a combination of these factors [32,33]. Increasing evidence indicates that many pediatric patients receiving anti-TNF agents are underdosed, highlighting the critical importance of TDM in optimizing efficacy and durability of response [8,34].

TDM involves the serial measurement of trough serum drug levels to guide dose optimization and maintain therapeutic concentrations. It can be applied in two main contexts:

- reactive TDM, performed in response to clinical deterioration or suspected LOR;
- proactive TDM, conducted at regular intervals from the initiation of therapy to preemptively adjust dosing and prevent treatment failure [35].

The ECCO–ESPGHAN guidelines now recommend early proactive TDM for all pediatric patients receiving anti-TNF therapy [12]. This approach is strongly supported by the PAILOT trial, a randomized controlled study of children with Crohn’s disease who initially responded to adalimumab. The trial demonstrated that proactive TDM resulted in significantly higher clinical remission rates compared with reactive TDM (82% vs. 48%), largely due to earlier dose intensifications in the proactive group [36].

For adalimumab, the first trough level measurement should be obtained immediately before the third injection, whereas for infliximab, trough levels should be checked prior to the fourth infusion [12]. In high-risk children—including those weighing <30 kg, with extensive disease, or hypoalbuminemia—TDM should be initiated earlier, typically before the second or third infusion [37].

According to ECCO–ESPGHAN recommendations, drug trough levels and ADA titers should be assessed at the end of induction in patients with primary LOR, and at the time of relapse in those with secondary LOR [38]. The results of TDM then guide individualized management strategies:

- low ADA titers, consider dose intensification and/or the addition of an immunomodulator;
- high ADA titers, recommend switching to an alternative anti-TNF agent (e.g., from infliximab to adalimumab) [39];
- low drug levels without ADA, proceed with dose escalation;
- therapeutic drug levels with ongoing LOR, switch to a biologic with a different mechanism of action.

These personalized, TDM-guided strategies have been shown to improve treatment efficacy, minimize immunogenicity, and prolong the durability of therapeutic response in PIBD [40].

Moreover, beyond pharmacokinetic optimization, there is growing recognition that TDM should be integrated with biomarker-based precision strategies to better individualize treatment in PIBD. Inflammatory biomarkers—including fecal calprotectin, C-reactive protein, serum cytokine profiles, and emerging microbiome-derived indicators—can complement TDM by providing real-time information on mucosal inflammation, risk of relapse, and likelihood of response to dose intensification. Combining pharmacokinetic (drug and antibody levels) and pharmacodynamic (biomarker) data supports a more holistic, personalized approach to therapeutic decision-making. Recent evidence from the reinforces this paradigm, demonstrating that individualized treatment adjustments guided by inflammatory biomarkers and proactive monitoring resulted in improved clinical and endoscopic outcomes compared with standard care, while reducing unnecessary treatment escalation [41]. These findings highlight the growing role of multimodal precision tools in optimizing biologic therapy and underscore the importance of integrating biomarker-based strategies into routine TDM algorithms for pediatric IBD.

### 3.5. Evidence of Safety

Anti-TNFα therapies are generally well tolerated in children; however, several adverse effects have been reported, including infusion-related reactions, opportunistic infections (notably *Mycobacterium tuberculosis*), dermatologic manifestations, and rare malignancies.

Infusion reactions are among the most frequent adverse events, particularly with infliximab. In the REACH trial, 17–18% of pediatric patients experienced infusion reactions, although only one case necessitated treatment discontinuation [42].

Infectious complications are a key safety concern, especially in children who are often treated concomitantly with immunomodulators or corticosteroids. Cohort studies evaluating infliximab and adalimumab have reported low rates of serious infections in pediatric patients [43]. In the REACH trial, serious infections occurred in 8% of cases, whereas a systematic review of 65 pediatric studies found no significant difference in the incidence of serious infections between patients receiving anti-TNF agents and those on immunomodulator monotherapy. Notably, infection rates were lower than in children treated with corticosteroids [44].

The risk of reactivating latent *Mycobacterium* infections is increased among children undergoing anti-TNF therapy, emphasizing the need for thorough tuberculosis (TB) screening before treatment initiation [39,45]. Anti-TNF agents are also associated with reactivation of chronic viral infections, including hepatitis B virus (HBV) and herpes zoster. Consequently, comprehensive pre-treatment screening is recommended in pediatric patients, encompassing:

- a detailed clinical history of TB exposure,
- tuberculin skin test (TST) and/or interferon-gamma release assay (IGRA),
- chest radiography, and
- serologic testing for HBV, HCV, and HIV [46].

Several studies have further evaluated TB risk in pediatric populations receiving biologic therapy. In a Spanish cohort of 221 children treated with infliximab or adalimumab, the prevalence of latent TB infection (LTBI) was 1.4%, with no incident cases of active TB during a median follow-up of 2.3 years, provided that screening (TST + IGRA) and chemoprophylaxis were appropriately performed [47]. Similarly, an integrative review involving approximately 36,000 pediatric rheumatic disease patients reported 80 cases of pulmonary TB associated with biologic use—mostly anti-TNFα agents—but the rate of active disease remained low when LTBI was treated prior to therapy [48]. A retrospective Turkish study of 73 pediatric patients on TNF inhibitors identified one case (1.3%) of active TB during follow-up, again underscoring the protective benefit of LTBI treatment before biologic initiation [49].

Prior to starting anti-TNFα therapy, vaccination status should be reviewed, and serologic testing performed to verify immunity. Booster doses may be necessary in non-immune patients. A preventive immunization strategy should include:

- annual influenza vaccination,
- pneumococcal vaccination,
- HPV vaccination, and
- hepatitis B vaccination for those lacking immunity [46,50].

Among dermatologic complications, infliximab-induced psoriasis (IIP) occurs in approximately 8–10% of pediatric patients. The condition affects both sexes equally and most commonly presents as non-pustular variants [51].

Concerns have also been raised regarding a potential association between anti-TNF therapy and malignancy, including Epstein–Barr virus–associated lymphomas, hemophagocytic lymphohistiocytosis, and hepatosplenic T-cell lymphoma. However, current evidence remains inconclusive. A systematic review by Dulai et al. found that the incidence of lymphoma among children treated with anti-TNF agents was comparable to that of the general pediatric population [44]. Similarly, Hyams et al. reported no increased malignancy risk among patients treated with infliximab compared with those not receiving biologic therapies [52].

## 4. Anti-Integrin Therapy in Pediatric Patients with Inflammatory Bowel Disease

Anti-integrin therapy represents a major advancement in the biologic management of IBD, offering a gut-selective mechanism that minimizes systemic immunosuppression. The therapeutic rationale for targeting integrins lies in their central role in mediating leukocyte trafficking across the intestinal endothelium, a key process in the perpetuation of mucosal inflammation characteristic of IBD [53,54,55]. Among available anti-integrin agents, vedolizumab (VDZ)—a humanized monoclonal antibody directed against the α4β7 integrin—has emerged as the most clinically relevant for both adult and pediatric populations. By specifically blocking the interaction between α4β7 integrin on lymphocytes and mucosal addressin cell adhesion molecule-1 (MAdCAM-1) on gut endothelial cells, VDZ effectively prevents lymphocyte migration into the gastrointestinal mucosa, thereby reducing intestinal inflammation while preserving systemic immune function [55,56,57]. Given its selective mechanism of action and favorable safety profile, VDZ has become the principal anti-integrin agent utilized in pediatric IBD. Its development was driven by the need for effective therapies capable of achieving mucosal healing while minimizing systemic adverse effects and infection risk—key considerations in long-term pediatric care [53,54,55,56,57].

Despite growing interest in VDZ as a gut-selective biologic with a favorable safety profile, several limitations constrain its application in PIBD. Most available evidence derives from small observational cohorts, retrospective series, or extrapolation from adult trials, resulting in limited high-quality, pediatric-specific data. Consequently, important clinical questions—such as optimal induction and maintenance dosing, the role of early therapy initiation, and long-term durability of response—remain incompletely answered. The comparatively slow onset of action of vedolizumab presents additional challenges in children with severe or rapidly progressive disease, often necessitating bridging therapies whose risks and benefits are not well defined in pediatric populations. Furthermore, while vedolizumab is approved for adults, its use in younger children frequently relies on off-label dosing strategies. Data on long-term safety, particularly regarding growth, pubertal development, infection risk, and the implications of prolonged integrin blockade, remain limited. The heterogeneity of disease phenotypes and prior biologic exposure across pediatric cohorts also complicates the interpretation of treatment effectiveness. Collectively, these gaps underscore the need for larger, prospective, multicenter pediatric studies to better define the therapeutic positioning, dosing strategies, and long-term outcomes of anti-integrin therapy in children with IBD.

### Vedolizumab

VDZ is a humanized monoclonal antibody with gut-selective activity that targets the α4β7 integrin, thereby inhibiting the migration of T lymphocytes into the inflamed intestinal mucosa [53]. This mechanism of action confers localized immune modulation, minimizing systemic immunosuppression. VDZ has demonstrated clinical efficacy in patients with IBD who are refractory to or intolerant of CS, immunomodulators, or anti-TNF agents [54]. It is effective in both CD and UC, although response rates tend to be higher in UC [55]. Notably, clinical outcomes are more favorable when VDZ is used as a first-line biologic, as its efficacy is reduced in patients previously exposed to anti-TNFα therapy [51].

Evidence regarding the use of VDZ in pediatric populations remains limited but encouraging. A systematic review of 10 studies including 455 children with CD or UC reported clinical remission (CR) rates of 48% at week 14 and 45% at one year in UC, and 28% at week 14 and 46% at one year in CD. Mucosal healing at one year was achieved in 15–34% of UC and 17–39% of CD cases [57].

For patients weighing ≥40 kg, VDZ is administered intravenously at 300 mg at weeks 0, 2, and 6 during induction, followed by maintenance infusions every 8 weeks. Although standardized pediatric dosing guidelines are lacking, younger children are often treated with an individualized dose of 6 mg/kg (up to a maximum of 300 mg) [58]. Because VDZ may require up to 16 weeks or longer to elicit a clinical response, some centers employ a short course of oral CS as bridging therapy during the induction phase [59].

Long-term data on the safety and durability of VDZ in children remain limited. However, the formation of ADA is rare [57,60], and serious adverse events necessitating discontinuation occur in only 5–10% of patients [61]. Importantly, VDZ use has not been associated with an increased risk of malignancy or opportunistic infections, supporting its favorable long-term safety profile [62,63].

Table 2 summarizes VDZ regimen in pediatric patients with IBD.

## 5. Anti-Interleukin Therapy in Pediatric Patients

Interleukin (IL) signaling plays a pivotal role in the pathogenesis of IBD, contributing to the dysregulated immune response that underlies chronic intestinal inflammation. Targeting key cytokines in the IL-12/IL-23 axis has emerged as a major therapeutic breakthrough, offering more selective modulation of immune pathways compared to traditional immunosuppressive or anti-TNF agents [64]. The shared p40 subunit of IL-12 and IL-23 is integral to the activation of Th1 and Th17 lymphocytes, both of which are strongly implicated in the inflammatory cascade characteristic of CD and UC [65]. The first biologic agent designed to inhibit this pathway was ustekinumab, a fully human monoclonal antibody that binds to the p40 subunit, thereby blocking the downstream signaling of both cytokines [64,65,66,67]. More recently, selective inhibitors of the IL-23 p19 subunit, including risankizumab and mirikizumab, have expanded the therapeutic landscape by offering even greater specificity and potentially improved safety and efficacy profiles [68,69,70,71,72,73,74,75]. Clinical trials in adults have demonstrated that these anti-IL agents are effective in inducing and maintaining remission in moderate-to-severe IBD, including patients with prior biologic failure. Emerging pediatric data, although still limited, suggest comparable efficacy and tolerability, positioning these agents as promising options in the management of refractory PIBD. Given their ability to selectively target key cytokine pathways involved in intestinal inflammation, anti-IL therapies represent a promising new frontier in the treatment of PIBD.

IL-targeted therapies share a common objective of modulating dysregulated immune pathways, yet they differ in selectivity, mechanism, and potential clinical advantages. Ustekinumab, which targets the shared IL-12/23 p40 subunit, provides broad inhibition of both Th1- and Th17-mediated inflammatory signaling, offering a well-established and effective option for patients with moderate-to-severe IBD. In contrast, newer selective IL-23 p19 inhibitors—risankizumab and mirikizumab—achieve more precise blockade of the IL-23–driven Th17 axis while sparing IL-12–dependent pathways, a distinction that may translate into improved mucosal healing, more durable remission, and a potentially enhanced safety profile. Early comparative data from adult clinical trials suggest that p19-selective agents may be particularly effective in biologic-experienced or refractory disease, whereas ustekinumab retains advantages in broader immunomodulation and longer-term safety experience. Although pediatric evidence remains limited, emerging real-world and early-phase pediatric studies indicate that all three agents demonstrate favorable tolerability and clinically meaningful remission rates, highlighting their growing importance in the management of refractory PIBD. Collectively, these distinctions underscore that anti-IL therapies represent an evolving therapeutic class in which increasing cytokine specificity may offer incremental benefits in efficacy, durability, and individualized treatment selection.

Although anti-interleukin therapies represent an important and rapidly expanding class of targeted biologics, several limitations constrain their current use in pediatric IBD. Most evidence supporting ustekinumab, risankizumab, and mirikizumab in children is extrapolated from adult clinical trials, with pediatric data largely limited to retrospective cohorts, small single-center experiences, or early-phase studies. As a result, key aspects of pediatric management—including optimal weight-based dosing, long-term safety, and the comparative effectiveness of p40 versus p19 inhibition—remain uncertain. Furthermore, all anti-IL agents aside from risankizumab in older adolescents are used off-label in the pediatric population, reflecting the absence of dedicated regulatory-approved dosing regimens for younger age groups. The heterogeneity of prior biologic exposure in available pediatric studies complicates interpretation of treatment response, and the relatively short follow-up periods limit insight into sustained remission, growth outcomes, immunogenicity, and rare adverse events. Additionally, while selective IL-23 inhibitors demonstrate promising efficacy in adult biologic-refractory disease, whether these advantages translate fully to children—particularly those with early-onset or complicated phenotypes—requires further investigation. These limitations highlight the urgent need for prospective, multicenter pediatric trials with standardized endpoints to better define the therapeutic role, safety profile, and long-term outcomes of anti-interleukin therapies in PIBD.

### 5.1. Mirikizumab

Mirikizumab is a humanized monoclonal antibody that selectively targets the p19 subunit of interleukin-23 (IL-23), thereby inhibiting downstream signaling pathways responsible for the overproduction of pro-inflammatory cytokines, particularly those secreted by T helper 17 (Th17) cells [64]. By modulating this pathway, mirikizumab aims to restore immune balance within the intestinal mucosa.

Although currently approved for adult patients with UC [65], mirikizumab has not yet received regulatory approval for pediatric use. Ongoing clinical trials are investigating its safety and efficacy in children and adolescents with moderate-to-severe UC who have shown an inadequate response to prior therapies, including CS, immunomodulators, or anti-TNF agents.

Preliminary results from the phase 2 SHINE 1 study have been encouraging, showing outcomes comparable to—or exceeding—those observed in adults from the LUCENT-1 trial [66]. In the pediatric cohort, the clinical response rate (based on the modified Mayo score) was 69.2% compared with 63.5% in adults, while clinical remission rates were 38.5% versus 25.6%, and endoscopic remission rates were 53.8% versus 36.3%, respectively [67]. Importantly, no new safety signals emerged in the pediatric population when compared with adult participants from LUCENT-1.

Most adverse events were mild to moderate, and no serious complications were reported during the 12-week induction phase. Pharmacokinetic analyses from the same trial identified weight-based dosing strategies to achieve optimal therapeutic exposure. The induction regimen consisted of 300 mg intravenous mirikizumab every four weeks (Q4W) for patients weighing >40 kg, and 5 mg/kg Q4W for those ≤40 kg. For maintenance therapy, subcutaneous administration was proposed at 200 mg Q4W for patients >40 kg, 100 mg Q4W for those >20 to ≤40 kg, and 50 mg Q4W for children ≤20 kg [68].

### 5.2. Risankizumab

Risankizumab is a humanized monoclonal antibody that selectively binds to the p19 subunit of IL-23, thereby inhibiting its interaction with the IL-23 receptor and suppressing downstream inflammatory pathways implicated in CD pathogenesis [69]. The drug has been approved for the treatment of CD in adults and in adolescents aged ≥16 years in certain regions, including Europe [66,70,71].

Emerging evidence suggests that risankizumab may represent a promising therapeutic option for pediatric patients with refractory CD, particularly those who have not achieved adequate disease control with conventional treatments or anti-TNF agents. Preliminary data indicate that risankizumab is effective in both inducing and maintaining remission, with a favorable safety profile.

In a retrospective analysis of 23 pediatric patients, steroid-free remission was achieved in 65%, clinical remission in 70%, and clinical response in 74% of cases [69,72]. Importantly, treatment efficacy was consistent across age groups, including children under 16 years of age, and no drug-related adverse events were reported during the observation period.

The dosing regimen used in these studies mirrored that established in adult protocols: an intravenous induction phase with 600 mg risankizumab administered at weeks 0, 4, and 8, followed by subcutaneous maintenance therapy consisting of 360 mg at week 12 and every 8 weeks thereafter [66,70,71].

Ongoing clinical trials, such as RisaKids, are currently evaluating risankizumab’s safety, efficacy, and pharmacokinetics in younger pediatric populations. These studies also aim to define weight-based dosing strategies and further establish risankizumab’s role in the treatment algorithm for pediatric CD [73].

### 5.3. Ustekinumab

Ustekinumab is a fully human monoclonal antibody that targets the p40 subunit shared by interleukin-12 (IL-12) and interleukin-23 (IL-23), thereby inhibiting both the Th1 and Th17 immune pathways implicated in the pathogenesis of UC. While ustekinumab is approved for adult UC, its use in children and adolescents remains off-label. Current ECCO–ESPGHAN guidelines recognize ustekinumab as a therapeutic option for moderate-to-severe UC refractory to conventional therapy and/or anti-TNF agents, although pediatric evidence remains limited compared with that for CD [74].

In adult clinical trials, ustekinumab demonstrated significant efficacy in both induction and maintenance of remission in UC [74]. Pediatric data, derived primarily from retrospective multicenter studies, also suggest meaningful clinical benefit. Reported clinical remission and steroid-free remission rates range from ~50% to 65% at one year, even among heavily pretreated populations [75]. An early clinical response (within 16 weeks) appears to predict long-term remission [76]. Improvements in inflammatory biomarkers (C-reactive protein, fecal calprotectin) and endoscopic healing have also been observed in subsets of pediatric patients [74].

The safety profile of ustekinumab in children with UC is generally favorable and consistent with adult experience. The most common adverse events are mild infections (such as nasopharyngitis or upper respiratory tract infections), headache, and injection-site reactions. Serious adverse events are uncommon, and there are no clear signals of malignancy or opportunistic infection to date. In one multicenter cohort, ustekinumab demonstrated both efficacy and safety in children with refractory UC and inflammatory bowel disease unclassified (IBDU) [75].

Nonetheless, potential risks must be carefully weighed. In that study, adverse events occurred in six children (10%), resulting in treatment discontinuation in three cases [75]. Despite this, ustekinumab offers several distinct advantages for pediatric UC management:

- a novel mechanism of action providing an alternative for patients who have failed anti-TNF therapy,
- low immunogenicity and a favorable tolerability profile, and
- infrequent subcutaneous maintenance dosing, which may improve adherence and quality of life [74].

However, its use in children remains off-label, and the evidence base is largely composed of small retrospective cohorts rather than prospective randomized trials. Uncertainties persist regarding long-term safety, particularly concerning growth, pubertal development, and sustained remission. Furthermore, cost and accessibility continue to pose challenges, especially in resource-limited settings [75].

Recent multicenter studies, including the ESPGHAN Porto Group cohort, reinforce ustekinumab’s short-term safety and efficacy in pediatric UC, while underscoring the urgent need for well-designed prospective trials to better define its long-term role in this population [75].

Table 3 summarizes data on the use of anti-IL therapies in pediatric patients with IBD.

## 6. The Newest Therapies Approved for Pediatric Population

**Figure 1:**
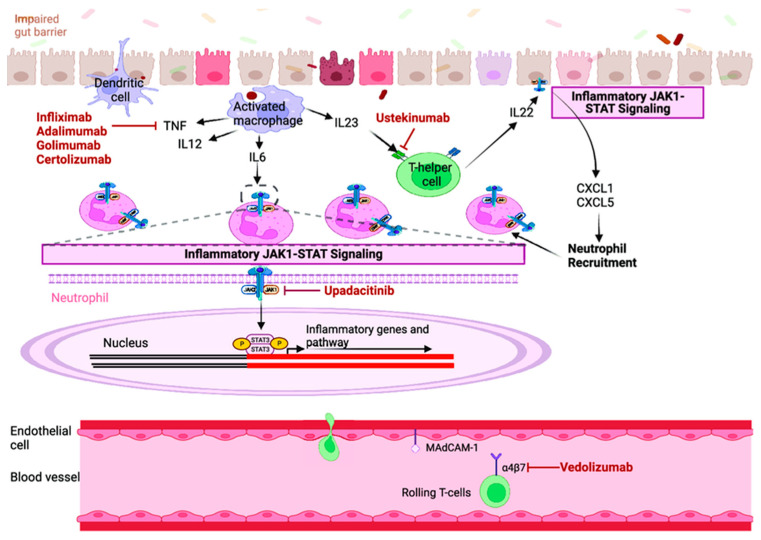
Mechanisms of action of advanced therapies targeting key inflammatory pathways in pediatric inflammatory bowel disease. Abbreviations: IL—Interleukin; IL-6—Interleukin-6; IL-12—Interleukin-12; IL-22—Interleukin-22; IL-23—Interleukin-23; JAK—Janus Kinase; JAK1—Janus Kinase-1; MAdCAM-1—Mucosal Addressin Cell Adhesion Molecule-1; STAT—Signal Transducer and Activator of Transcription; STAT3—Signal Transducer and Activator of Transcription-3; TNF—Tumor Necrosis Factor; α4β7—Alpha-4 Beta-7 Integrin.

Figure 1 illustrates the major immunological pathways targeted by advanced therapies in PIBD.

This schematic illustrates the major immune pathways implicated in gut inflammation and the corresponding therapeutic targets of biologics and small-molecule agents. Damage to the intestinal epithelial barrier permits antigen presentation by dendritic cells and activation of macrophages, leading to the production of pro-inflammatory cytokines including TNF, IL-12, IL-23, and IL-6. Anti-TNF agents (infliximab, adalimumab, golimumab, certolizumab) block TNF-mediated signaling and downstream activation of inflammatory cascades. Ustekinumab inhibits the IL-12/23 pathway by binding the shared p40 subunit, thereby reducing Th1 and Th17 cell activation and limiting IL-22–driven inflammation. IL-6 and other cytokines activate the JAK-STAT pathway, promoting transcription of pro-inflammatory genes; the JAK1-selective inhibitor upadacitinib interrupts this intracellular signaling cascade to reduce cytokine-mediated inflammation. Chemokine-mediated recruitment of neutrophils (e.g., through CXCL1 and CXCL5) further amplifies mucosal injury. Vedolizumab selectively blocks α4β7 integrin on circulating T cells, preventing their adhesion to MAdCAM-1 on gut endothelial cells and thereby reducing lymphocyte trafficking to the intestinal mucosa. Together, these therapeutic mechanisms target complementary aspects of the immune response contributing to chronic intestinal inflammation.

Traditional therapies for IBD—including 5-ASA and immunomodulators—remain important components of disease management, particularly during acute disease phases. However, these treatments have well-recognized limitations, such as modest long-term efficacy and safety concerns related to the broad immunosuppressive effects of conventional agents.

The advent of anti-TNFα therapies represented a major milestone in IBD management, offering a more targeted approach to inflammation control. Despite their transformative impact, anti-TNF monoclonal antibodies are hindered by high rates of primary non-response and secondary loss of response over time [77].

In response to these limitations, novel therapeutic strategies have emerged, including Janus kinase (JAK) inhibitors and sphingosine-1-phosphate (S1P) receptor modulators, which act by modulating cytokine signaling or immune-cell trafficking. These small-molecule therapies offer distinct advantages over biologics, such as oral administration, absence of immunogenicity, and shorter washout periods, making them attractive alternatives for certain patient populations.

Over the past two decades, growing evidence has shown that many cytokines involved in autoimmune and inflammatory diseases signal through the JAK–STAT pathway. Accordingly, both pan-JAK inhibitors and selective JAK inhibitors have been developed as a new class of targeted therapies, designed to modulate cytokine-mediated immune responses across a range of immune-mediated inflammatory diseases [78].

Despite the growing interest in JAK inhibitors and S1P modulators as promising oral, rapidly acting therapies for refractory pediatric IBD, several important limitations must be acknowledged. Most available evidence in children consists of small retrospective cohorts, compassionate-use experiences, and early real-world series, with no large randomized pediatric trials currently available. As a result, the safety, efficacy, and optimal dosing strategies of these small-molecule agents remain incompletely characterized in younger populations. Their use in children—especially those under 16 years—remains predominantly off-label, and weight-based pharmacokinetic data are sparse. Concerns regarding potential class-specific risks, including serious infections, thromboembolic events, lipid abnormalities, hepatic toxicity, and rare immunologic complications, are based almost entirely on adult studies and may not fully translate to pediatric physiology. Additionally, the long-term consequences of modulating broad cytokine pathways (JAK inhibitors) or lymphocyte trafficking (S1P modulators) during critical stages of growth and immune development remain unknown. Heterogeneity in induction regimens, concomitant corticosteroid use, and previous biologic failure across published pediatric cohorts further complicates assessment of true treatment effectiveness. Taken together, these limitations underscore the need for rigorous, prospective, multicenter pediatric studies to define the long-term safety, efficacy, and optimal positioning of small-molecule therapies in the evolving treatment algorithm for PIBD.

### 6.1. JAK-Inhibitors

The management of PIBD has undergone a profound transformation over the past decade. Alongside conventional therapies, a growing number of advanced biologics—including vedolizumab, ustekinumab, and risankizumab—and small molecules such as tofacitinib, etrasimod, and upadacitinib have expanded the therapeutic armamentarium. Increasingly, personalized medicine approaches—integrating precision-based therapy selection, TDM, and lifestyle optimization—are shaping modern PIBD management [79].

A pivotal discovery in recent decades has been the recognition that numerous cytokines implicated in autoimmune and inflammatory disorders signal through the Janus kinase–signal transducer and activator of transcription (JAK–STAT) pathway. Four JAK have been identified—JAK1, JAK2, JAK3, and tyrosine kinase 2 (TYK2)—each with distinct but overlapping roles in immune regulation. More recently, a new class of therapies—JAK inhibitors—has emerged, encompassing both pan-JAK and selective JAK inhibitors, designed to modulate downstream cytokine signaling across various immune-mediated inflammatory diseases. Among these agents, JAK inhibitors (or “small molecules”) represent the first oral therapeutic class approved for the treatment of IBD. Their advantages include a rapid onset of action, short plasma half-life, and lack of immunogenicity, making them attractive alternatives to biologic therapies [80].

Tofacitinib is a non-selective JAK inhibitor targeting JAK1, JAK2, JAK3, and TYK2, thereby interrupting cytokine-driven intracellular signaling and immune activation [81]. It was the first small molecule approved by the U.S. Food and Drug Administration (FDA) and European Medicines Agency (EMA) for the treatment of moderate-to-severe UC in adults. Its efficacy and safety were established in three pivotal phase 3 trials—OCTAVE-1, OCTAVE-2, and OCTAVE Sustain—which confirmed its benefit as both induction and maintenance therapy [81].

In children, however, clinical experience remains limited, and use is restricted to off-label cases refractory to other therapies, including anti-TNFα agents and vedolizumab. Initial evidence came from three small single-center studies, the largest of which involved 21 pediatric patients. More recent data from a retrospective study at the Children’s Hospital of Philadelphia evaluated tofacitinib in patients ≤21 years (median age 14.4 years at diagnosis, 18.4 years at treatment). Most participants had failed multiple biologic therapies, and over two-thirds were receiving corticosteroids at treatment initiation [82].

In this cohort, clinical improvement was observed by week 6, with nearly 50% achieving a clinical response, and efficacy comparable to adult outcomes, even in patients with refractory disease. These findings suggest that tofacitinib may represent a viable off-label option for pediatric UC patients refractory to anti-TNF therapy who require rapid disease control [82].

Further support comes from the largest multicenter observational study to date, which evaluated 101 pediatric UC patients (aged 2–18 years) across 16 centers in Europe, North America, and the Middle East, followed for a median of 24 weeks [83]. Most children were highly refractory, with 75% having failed ≥2 biologics and a median PUCAI score of 50 at baseline. At treatment start, 60 patients were on CS, and 10 received combination therapy with another biologic (5 vedolizumab, 3 ustekinumab, 2 infliximab, 1 adalimumab).

At week 8, 16% achieved CS-free remission, increasing to 23% by week 24, while 19% achieved deep remission (defined as symptom resolution and fecal calprotectin < 100 µg/g) [78]. Although these results are promising, interpretation is limited by concomitant therapies and heterogeneous induction regimens. No predictive factors of response (age, phenotype, duration, or severity) were identified, mirroring findings in adult studies [84], though lower baseline disease activity appears associated with improved outcomes [85].

Safety findings were reassuring. No serious adverse events, allergic reactions, or treatment discontinuations were reported. A single herpes zoster infection was possibly treatment-related, while minor changes in lipid and liver enzyme levels were clinically insignificant [83]. Overall, the pediatric safety profile aligns closely with adult experience, supporting tofacitinib as a potential therapeutic alternative for refractory pediatric UC, warranting further prospective evaluation [83].

Upadacitinib is a selective JAK1 inhibitor approved by the FDA, EMA, and other regulatory agencies for a range of chronic immune-mediated inflammatory diseases, including rheumatologic, dermatologic, and gastrointestinal conditions [86]. It is currently the only small molecule approved for both UC and CD, with a favorable benefit–risk profile demonstrated across multiple phase III clinical trials. In the U-ACHIEVE study, 45 mg upadacitinib induced clinical remission in 19.6% of adult UC patients by week 8 [87].

In pediatrics, available data remain limited but encouraging. A single-center study of 12 children with refractory IBD reported clinical remission in 92% (11/12) between weeks 8 and 12 [88]. Additionally, a multicenter cohort study involving six European ESPGHAN centers and one North American site evaluated upadacitinib as salvage therapy for acute severe UC (ASC) (PUCAI ≥ 65) [89]. Most patients had failed at least one biologic, and 73% had failed two or more (including infliximab, vedolizumab, ustekinumab, and tofacitinib). Treatment was well tolerated, with no discontinuations due to AEs. Mild infections—one herpes simplex virus and one cytomegalovirus (CMV) colitis—and hyperlipidemia were the most frequent adverse events, all occurring in patients concurrently on corticosteroids [89].

No thromboembolic events were observed, though vigilance remains critical given the elevated baseline risk of venous thromboembolism in ASC. Anticoagulant prophylaxis may be appropriate in high-risk cases [89]. These results are consistent with adult studies, including a Chinese cohort of 14 adults with ASC, all of whom achieved complete clinical response, with only one colectomy [90].

Upadacitinib was administered in various regimens—alone, with CS, or with previously failed biologics—but no differences in efficacy were detected between these strategies [91]. Collectively, these findings support upadacitinib as a promising sequential therapy for pediatric patients with steroid- and infliximab-refractory ASC, including those unresponsive to tofacitinib [91].

Long-term adult data reinforce its potential as maintenance therapy: in a systematic review, 80% of patients maintained steroid-free remission over 4–24 weeks [92]. Thus, upadacitinib may serve as both an induction and maintenance option, potentially reducing the need for surgery and improving long-term quality of life in pediatric UC [92].

Compared with biologic therapies, JAK inhibitors offer several practical and mechanistic advantages that make them an attractive option in the management of refractory PIBD. As oral small molecules, they eliminate the need for intravenous infusions or subcutaneous injections, improving convenience and potentially enhancing adherence, especially in adolescents. Their rapid onset of action—often within days—contrasts with the slower therapeutic effect observed with many biologics, making JAK inhibitors particularly useful in acute or rapidly progressive disease. Additionally, small molecules lack immunogenicity, avoiding the development of anti-drug antibodies that can compromise the durability of biologic therapy and necessitate dose escalation or switching. Their short half-life also allows faster discontinuation in cases of adverse events, infections, or treatment failure, offering greater flexibility than biologics, which often require prolonged washout periods. From a mechanistic perspective, JAK inhibitors target multiple cytokine pathways simultaneously, potentially overcoming inflammatory redundancy that limits the effectiveness of single-cytokine–targeting biologics. Although long-term safety in children requires further investigation, these comparative advantages position JAK inhibitors as valuable alternatives or adjuncts for patients who have failed conventional therapies and multiple biologic classes.

### 6.2. S1P Modulators

The disruption of lymphocyte trafficking and recruitment within the intestinal tract is a central mechanism in the pathogenesis of IBD. Aberrant cytokine production exposes lymphocytes to chemotactic signals, predominantly chemokines, which activate integrins on their surface. This activation promotes the targeted migration and accumulation of lymphocytes within the intestinal mucosa, particularly at sites of inflammation, thereby perpetuating chronic immune activation and tissue injury [93].

S1P is a bioactive sphingolipid that, through binding to its five known receptors (S1PR1–S1PR5), regulates diverse physiological and pathological processes across multiple organ systems, including the immune, nervous, and cardiovascular systems. Its pivotal role in immune-cell trafficking, particularly the egress of T lymphocytes from secondary lymphoid organs, has led to the development of S1P receptor modulators. These agents function by sequestering subsets of T cells within lymphoid tissues, preventing their migration to inflamed intestinal sites and thereby attenuating mucosal inflammation. Through this targeted mechanism, S1P modulators represent a novel therapeutic approach in IBD management [94].

The contribution of S1P receptors (S1PRs) to IBD pathogenesis has been demonstrated in both preclinical and clinical studies. In dextran sodium sulfate (DSS)-induced colitis models, modulation of the S1P signaling pathway was shown to influence intestinal inflammation [95]. Similarly, gene expression analyses of colonic biopsy samples from patients with acute UC revealed significant dysregulation of S1P-related pathways [96]. In PIBD cohorts, patients with active moderate-to-severe disease exhibited upregulated expression of S1P-related genes compared with both healthy controls and patients in remission [97].

Several S1P receptor modulators have since been developed for immune-mediated disorders. Early-generation non-selective prodrugs, such as fingolimod, were first approved for relapsing-remitting multiple sclerosis, but newer, more selective agents with improved safety profiles—namely ozanimod (not currently available in Italy) and etrasimod—have gained approval for the treatment of moderate-to-severe UC [97].

Etrasimod is a selective modulator of S1P receptors, designed to reduce lymphocyte migration to the intestinal mucosa and thereby dampen local inflammation. This results in measurable clinical improvements, including reduced rectal bleeding, decreased stool frequency, and enhanced mucosal healing, culminating in higher rates of clinical remission [98].

In the phase II OASIS trial, etrasimod demonstrated significant short-term efficacy, inducing both clinical and endoscopic improvement at 12 weeks, with benefits largely sustained for up to one year in extension studies [99]. Approximately 60% of patients experienced at least one adverse event—mostly mild or moderate—including worsening UC, anemia, headache, upper respiratory tract infections, and transient elevations in liver enzymes. The expected reversible lymphopenia confirmed its pharmacodynamic mechanism, with no significant increase in serious infections observed [99].

Building on these findings, two phase III randomized controlled trials, ELEVATE UC 12 and ELEVATE UC 52, evaluated etrasimod in a broader adult population with moderate-to-severe UC, including patients with isolated proctitis. Participants received etrasimod 2 mg or placebo, with rigorous safety monitoring (hematology, ECG, pulmonary, and ophthalmologic assessments) [100]. Both studies demonstrated superiority of etrasimod over placebo, achieving significantly higher clinical remission rates at both induction and maintenance phases. Collectively, these trials confirmed a favorable safety profile, with manageable adverse effects and sustained therapeutic benefit over time [101].

S1P modulators thus represent an important addition to the therapeutic landscape of IBD, offering key advantages such as short washout periods, reversible immunosuppression, and oral administration—factors that may enhance treatment adherence and patient quality of life [77].

The approval of etrasimod by the EMA marks a significant milestone, establishing it as a safe and effective treatment for adolescents aged ≥16 years with moderate-to-severe UC. Its gut-selective mechanism of action distinguishes it from anti-TNFα agents and other biologics, while its oral route of administration is particularly appealing for younger patients, improving both treatment convenience and compliance [100].

Table 4 summarizes data on the use of JAK inhibitors and S1P modulators in pediatric patients with IBD.

## 7. Knowledge Gaps

Recent studies [102,103,104] further illustrate both the therapeutic potential and the persistent uncertainties in the management of PIBD. These reports—among the first to provide real-world pediatric data on advanced therapies—highlight that (i) response rates vary considerably according to prior treatment exposure, baseline disease severity, and concomitant medications; (ii) long-term safety data remain sparse; and (iii) a substantial proportion of children still require dose escalation, treatment intensification, or therapeutic switching over time, underscoring that neither biologic nor small-molecule therapy guarantees durable remission in all cases. Moreover, regional differences in drug availability, regulatory approval, and reimbursement policies contribute to marked heterogeneity in treatment initiation, maintenance strategies, and follow-up practices. Collectively, these findings reinforce the need for well-designed pediatric-specific prospective trials, long-term registries, and harmonized treatment protocols—particularly to define predictors of response, age- and weight-appropriate dosing, and long-term safety across diverse healthcare settings.

Despite substantial therapeutic advances, several critical knowledge gaps continue to limit the precision, equity, and long-term effectiveness of PIBD management. One major unresolved issue concerns the optimal timing of biologic initiation. Although early biologic (“top-down”) therapy may reduce cumulative inflammatory burden, promote mucosal healing, and potentially modify disease trajectory, evidence supporting this approach in children remains limited. Most pediatric data derive from retrospective cohorts or extrapolation from adult studies, and the comparative benefit of early biologic intervention versus traditional step-up strategies has not been definitively established. Identifying which children are most likely to benefit from early intensive therapy—and defining the phenotypic, genetic, or biomarker profiles that predict such benefit—remains a major unmet research priority.

### 7.1. Safety-Related Knowledge Gaps: Mechanisms and Class-Specific Differences

Important safety-related gaps persist, particularly regarding the long-term consequences of immune modulation during childhood and adolescence, a critical period for growth, pubertal development, and immune maturation. While biologic therapies generally offer a more targeted mechanism of action than systemic corticosteroids, their cumulative immunosuppressive effects—especially when used long term or in combination regimens—are incompletely characterized in pediatric populations. Anti-TNF agents primarily suppress TNF-mediated inflammatory signaling, whereas anti-integrin and anti-interleukin therapies exert more selective effects on leukocyte trafficking or cytokine pathways. These mechanistic differences likely translate into distinct safety profiles, yet comparative long-term pediatric data are lacking.

Small-molecule therapies introduce additional safety uncertainties. JAK inhibitors, by broadly interfering with intracellular cytokine signaling, may affect multiple immune and metabolic pathways simultaneously. Adult data have raised concerns regarding thromboembolic events, herpes zoster reactivation, lipid abnormalities, and laboratory perturbations, but pediatric-specific risk estimates remain poorly defined. Similarly, S1P receptor modulators alter lymphocyte trafficking through receptor-mediated sequestration in lymphoid tissues, resulting in predictable lymphopenia; however, the long-term implications of sustained lymphocyte redistribution during immune system development are unknown. Importantly, most pediatric evidence for these agents derives from small, heterogeneous cohorts with limited follow-up, precluding robust assessment of rare or delayed adverse events.

### 7.2. Growth and Development: Biologics Versus Corticosteroids

A particularly important and underexplored issue is the comparative impact of advanced therapies versus corticosteroids on growth and pubertal development. Corticosteroids are well known to impair linear growth, delay puberty, reduce bone mineral density, and disrupt metabolic homeostasis in children. In contrast, biologic therapies—by achieving sustained inflammation control and enabling steroid-sparing strategies—have been associated with improved growth velocity and catch-up growth in several pediatric cohorts. However, whether different biologic classes confer equivalent benefits on growth outcomes remains unclear, and data directly comparing biologics, small molecules, and prolonged corticosteroid exposure are scarce. Furthermore, the long-term developmental consequences of initiating immune-targeted therapies early in life—particularly small molecules with broader intracellular effects—remain insufficiently studied.

### 7.3. Cost-Effectiveness and Access

Cost-effectiveness represents another major unresolved dimension of PIBD care. Although biologics and small-molecule therapies provide substantial clinical benefit, they are associated with high direct and indirect costs, raising questions about sustainable resource allocation in both high-income and resource-limited settings. Pediatric-specific economic evaluations are rare and complicated by unique considerations, including growth outcomes, long-term disease burden, transition to adult care, and lifetime healthcare utilization. While biosimilars offer a promising opportunity to reduce costs and expand access, their adoption remains uneven due to regulatory, reimbursement, and cultural barriers. Robust pediatric-focused cost-effectiveness analyses are urgently needed to inform policymakers and healthcare systems.

### 7.4. Biomarkers and Precision Medicine

Another key knowledge gap concerns the integration of biomarkers into routine pediatric clinical decision-making. Tools such as fecal calprotectin, therapeutic drug monitoring, and emerging genomic and transcriptomic markers hold promise for predicting treatment response, individualizing dosing, and monitoring subclinical inflammation. However, standardized pediatric thresholds, optimal sampling intervals, and the comparative utility of different biomarkers remain poorly defined. Moreover, it is unclear how these tools should be combined into multimodal predictive models capable of guiding individualized therapeutic strategies. Prospective pediatric studies evaluating biomarker-driven treatment algorithms—including dashboard-based or algorithmic decision support—represent a critical research priority.

### 7.5. Global Disparities

Finally, global disparities in access to advanced therapies continue to undermine equitable PIBD care. In low- and middle-income countries, limited drug availability, delayed diagnosis, restricted access to pediatric gastroenterology specialists, and inconsistent insurance coverage contribute to suboptimal outcomes. Even where biosimilars are available, infrastructure for safe administration, laboratory monitoring, and therapeutic drug monitoring is often inadequate. As a result, treatment decisions are frequently dictated by economic and logistical constraints rather than clinical need. Addressing these disparities will require coordinated global advocacy, enhanced international guideline harmonization, and targeted investment in pediatric IBD infrastructure.

Collectively, these knowledge gaps underscore the urgent need for multicenter pediatric trials, long-term safety registries, improved economic analyses, and international strategies to promote equitable access. Advances in precision medicine must be matched by parallel efforts to ensure that all children—regardless of geography or socioeconomic status—can safely benefit from the rapidly evolving therapeutic landscape in inflammatory bowel disease.

## 8. Conclusions

The management of PIBD has evolved dramatically over the past two decades, shifting from traditional reliance on CS and immunomodulators to the widespread use of biologic agents and, more recently, small-molecule therapies. Among biologics, anti-TNF agents—particularly infliximab and adalimumab—remain the cornerstone of therapy, with proven efficacy in inducing and maintaining remission, promoting mucosal healing, and improving both growth outcomes and quality of life. The advent of TDM has further advanced personalized care by optimizing dosing, reducing immunogenicity, and minimizing the risk of loss of response.

The emergence of next-generation biologics—including vedolizumab, ustekinumab, risankizumab, and mirikizumab—has expanded treatment options for children who are refractory to or intolerant to anti-TNF therapy. Preliminary evidence suggests promising efficacy and favorable safety profiles, though long-term pediatric data remain limited. Parallel progress in small-molecule therapies, such as JAK inhibitors (tofacitinib, upadacitinib) and S1P modulators (etrasimod), represents an important therapeutic advance, particularly for severe, treatment-resistant disease. However, their off-label use in children necessitates careful risk–benefit assessment and close safety monitoring.

While substantial therapeutic advances have transformed the management of PIBD, important gaps in knowledge continue to limit the precision and equity of care. The evidence base remains dominated by retrospective studies and extrapolated adult data, with a persistent scarcity of pediatric randomized controlled trials to guide age-specific dosing, sequencing of advanced therapies, and long-term safety surveillance. Interpretation of current efficacy and safety outcomes is further complicated by heterogeneity in study populations, including wide variation in patient age, baseline disease severity, previous biologic exposure, and the off-label dosing strategies often required in younger children. These factors make cross-study comparisons challenging and may obscure true treatment effects. In addition, regional differences in access to biologics, biosimilars, and emerging small-molecule therapies create disparities in care that disproportionately affect children in low-resource settings. Addressing these limitations will require coordinated, multicenter pediatric trials, harmonized dosing and monitoring strategies, and policies aimed at improving global access to advanced therapies. Continued integration of pharmacogenomics, therapeutic drug monitoring, and biomarker-driven approaches will be essential to achieve a more personalized, evidence-based, and equitable treatment paradigm for all children living with IBD.

In summary, this review provides a comprehensive and clinically integrated synthesis of the rapidly evolving therapeutic landscape in PIBD, uniquely bridging mechanistic insights, evidence-based treatment positioning, and emerging precision-medicine strategies. By consolidating current data on biologics, small molecules, biomarker-guided monitoring, and real-world challenges in access and implementation, the review offers clinicians a practical framework for navigating increasingly complex treatment decisions. At the same time, it identifies critical gaps in pediatric-specific evidence and underscores the urgent need for harmonized global research efforts. Together, these elements position the review as a valuable resource for practitioners and researchers seeking to optimize, personalize, and expand the therapeutic opportunities available to children living with IBD.

## Figures and Tables

**Table 1:** Anti-TNF therapy approved for pediatric patients with IBD.

Agent	Type of Antibody	Administration	Mechanism of Action	Pediatric Dosing Scheme
Infliximab	Chimeric IgG (human/murine)	IV infusion	Binds soluble and membrane-bound TNFα, blocking receptor interaction	≥30 kg: 5 mg/kg IV at weeks 0, 2, 6 (induction), then q8w (maintenance). <30 kg or severe disease: 10 mg/kg or shorter intervals may be required
Adalimumab	Fully human IgG	SC injection	Same as infliximab	≥40 kg: 160 mg at week 0, 80 mg at week 2, then 40 mg q2w (maintenance). <40 kg: 80 mg at week 0, 40 mg at week 2, then 20 mg q2w (maintenance)

**Table 2:** Vedolizumab therapy in pediatric patients with inflammatory bowel disease.

Agent	Type of Antibody	Administration	Mechanism of Action	Pediatric Dosing Scheme
Vedolizumab	Humanized IgG1 monoclonal antibody	IV infusion	Targets α4β7 integrin, blocking T-cell migration into inflamed gut mucosa	≥40 kg: 300 mg IV at weeks 0, 2, 6 (induction), then q8w (maintenance). <40 kg: Individualized dosing ~6 mg/kg (max 300 mg). Often combined with a short course of oral corticosteroids as bridging therapy during induction.

**Table 3:** Anti-interleukin therapies in pediatric patients with inflammatory bowel disease.

Agent	Target	Administration	Pediatric Evidence	Pediatric Dosing Scheme *
Mirikizumab	IL-23 (p19 subunit)	IV induction, then SC maintenance	Phase 2 SHINE-1 study: promising response/remission, safety similar to adults	>40 kg: 300 mg IV q4w (induction); maintenance SC: 200 mg q4w. ≤40 kg: 5 mg/kg IV (induction); SC: 50–100 mg q4w depending on weight
Risankizumab	IL-23 (p19 subunit)	IV induction, then SC maintenance	Retrospective pediatric data: ~65% steroid-free remission; ongoing RisaKids trial	Induction: 600 mg IV at weeks 0, 4, 8. Maintenance: 360 mg SC q8w
Ustekinumab	IL-12/23 (p40 subunit)	IV induction, then SC maintenance	Retrospective pediatric cohorts: ~50–65% remission at 1 year; safety favorable	Adult-like dosing; weight-based IV induction followed by SC maintenance (off-label in children)

* Dosing regimens are largely extrapolated from adult protocols; pediatric use is often off-label and requires individualized adjustment.

**Table 4:** JAK inhibitors and S1P modulators in pediatric patients with inflammatory bowel disease.

Agent	Class/Target	Administration	Pediatric Evidence and Indication	Pediatric Dosing Scheme *
Tofacitinib	Pan-JAK inhibitor (JAK1/2/3, TYK2)	Oral (tablet)	Limited pediatric data (retrospective multicenter study, n = 101); used off-label for refractory UC after ≥2 biologics. Rapid onset by week 6; similar response rates to adults.	Off-label use. Adult regimen extrapolated: Induction 10 mg BID; Maintenance 5–10 mg BID, individualized. Close monitoring required.
Upadacitinib	Selective JAK1 inhibitor	Oral (tablet)	Pediatric use reported in small cohorts (n = 12, ASC cases). Clinical remission 92% at weeks 8–12. Salvage therapy after biologic failure; favorable safety profile.	Adult-like regimen extrapolated: Induction 45 mg QD (8–12 weeks); Maintenance 15–30 mg QD. Adjust based on response and safety.
Etrasimod	S1P receptor modulator	Oral (tablet)	EMA-approved for UC ≥ 16 years. Pediatric data limited but promising. Gut-selective mechanism; good safety profile.	Adult regimen: 2 mg QD. Pediatric dosing not yet standardized; use in ≥16 years according to EMA approval.

* Dosing regimens are largely extrapolated from adult protocols; pediatric use is often off-label and requires individualized adjustment.

## Data Availability

No new data were created or analyzed in this study. Data sharing is not applicable.
